# Improvement of the Pharmacological Properties of Maize RIP by Cysteine-Specific PEGylation

**DOI:** 10.3390/toxins8100298

**Published:** 2016-10-17

**Authors:** Ka-Yee Au, Wei-Wei Shi, Shuai Qian, Zhong Zuo, Pang-Chui Shaw

**Affiliations:** 1 Centre for Protein Science and Crystallography, School of Life Sciences, The Chinese University of Hong Kong, Shatin, N.T., Hong Kong, China; aaky73@yahoo.com.hk (K.-Y.A.); Shiww@cuhk.edu.hk (W.-W.S.); 2 School of Pharmacy, Faculty of Medicine, The Chinese University of Hong Kong, Shatin, N.T., Hong Kong, China; silence_qs@163.com (S.Q.); joanzuo@cuhk.edu.hk (Z.Z.)

**Keywords:** MOD, PEGylation, antigenicity, pharmacokinetics study, circulation half-life, antibody induction

## Abstract

To improve the pharmacological properties of maize ribosome-inactivating protein (maize RIP) for targeting HIV-infected cells, the previously engineered TAT-fused active form of maize RIP (MOD) was further engineered for cysteine-directed PEGylation. In this work, two potential antigenic sites, namely Lys-78 and Lys-264, were identified. They were mutated to cysteine residue and conjugated with PEG_5k_ or PEG_20k_. The resultant PEG derivatives of MOD variants were examined for ribosome-inactivating activity, circulating half-life and immunogenicity. Our results showed that MOD-PEG conjugates had two- to five-fold lower biological activity compared to the wild-type. Mutation of the two sites respectively did not decrease the anti-MOD IgG and IgE level in mice, but the conjugation of PEG did dramatically reduce the antigenicity. Furthermore, pharmacokinetics studies demonstrated that attachment of PEG_20k_ prolonged the plasma half-life by five-fold for MOD-K78C and 17-fold for MOD-K264C, respectively. The site-specific mutation together with PEGylation therefore generated MOD derivatives with improved pharmacological properties.

## 1. Introduction

Ribosome-inactivating proteins (RIPs) are RNA *N*-glycosidases that remove a specific adenine at the α-sarcin/ricin loop (SRL) of large ribosomal RNA (rRNA) and in turn cease protein synthesis [1,2]. 

RIPs are categorized into three types according to their structural organization. Type 1, represented by trichosanthin (TCS) [3] and pokeweed antiviral protein (PAP) [4], is a monomeric protein with full SRL depurinating activity. Type 2, such as ricin and abrin [5], is heterodimeric with an enzymatically active A chain linked to a lectin-like B chain by a disulphide bond. Maize RIP is a type 3 RIP, which is first synthesized with an extra 25-amino-acid internal segment and requires proteolytic removal upon which the precursor is activated to resume *N*-glycosidase function [6,7]. We have recently fused the HIV-1 TAT transduction peptide to the *N*-termini of maize RIP and exploited its unique activity regulatory feature of maturation by introducing HIV-1 protease recognition sequences to the internal inactivation region to sensitize maize RIP towards HIV-infected cells, in which the activation of maize RIP was triggered by HIV-1 protease [8]. Our work has demonstrated the potential inhibitory effect of maize RIP towards immunodeficiency viruses, both in HIV-infected cells [8] and in Chinese rhesus macaques [9], suggesting its potential as a novel anti-HIV agent.

The therapeutic applications of RIPs, however, have long been challenged for two reasons. First, since most RIPs are naturally produced in plants, their uses in animals are prone to trigger adverse immune responses. AIDS patients receiving PAP-derived immunotoxin showed undesirable antibody production targeting the cytotoxin moiety [10] and patients infused with trichosanthin were found to develop allergic symptoms with TCS-specific antibodies detected in vivo, and repeated administration of TCS might cause fetal anaphylaxis [11]. Second, proteins of low molecular weight are susceptible to rapid elimination from the circulation through renal filtration or degradation [12]. Typically, RIPs are of sizes ranging from 27 to 32 kDa and the 27 kDa trichosanthin was previously reported to have a plasma residence time between 8.4 and 12.7 min [13]. The short circulation retention necessitates frequent infusion to maintain an effective concentration for treatment and decreases the medical value of RIPs. 

Various strategies have been adopted to improve the aforementioned shortfalls and PEGylation, and the covalent coupling with polymer polyethylene glycol (PEG) is the most common. With high hydrophilicity and low immunogenicity, PEG attachment increases the size of biopharmaceuticals for a long circulating life as well as confers a shielding effect at antigenic sites for low immune responses [14,15]. PEG-adenosine deaminase (ADA) is the first FDA-approved drug modified by PEGylation and its clinical application for treating ADA deficiency is positive and well tolerated [16]. We have coupled Icarapin, a bee venom protein, with PEG in a site-specific manner to diminish antibody generation [17], and also mono-PEGylated trichosanthin to slow down the circulation clearance and decrease the immunogenicity [18,19].

In this study, we aim to enhance the therapeutic value of TAT-fused maize RIP through conjugation with PEG. Our results demonstrated the resultant PEGylated MOD, especially PEG_20k_-conjugated MOD shows a prolonged circulating half-life in plasma and decreased immunogenicity, while only lost half of the ribosome-inactivating activity. Our study will benefit therapeutic applications of MOD to combat HIV/AIDS and the development of effective RIP-related biomedicine as therapeutics.

## 2. Results

### 2.1. Selection of PEGylation Sites

**Figure 1:**
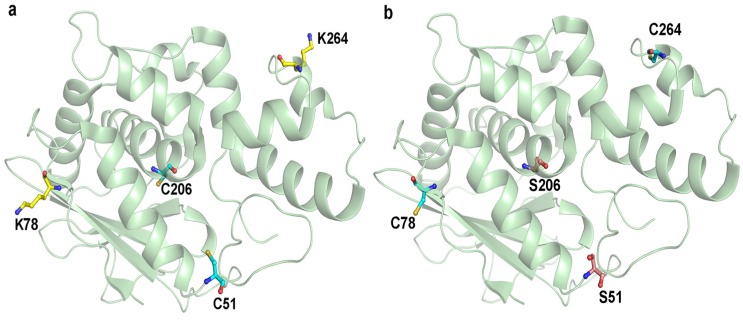
MOD (**a**) before and (**b**) after mutations. Cysteine, lysine and serine residues were highlighted with cyan, yellow and pink colors, respectively. C51 and C206 were mutated to serine and the selected PEGylated sites, K78 and K264, were mutated to cysteine for PEGylation.

This study aimed to improve the therapeutic potential of maize RIP by cysteine-directed PEGylation and the conjugation sites were chosen based on three criteria: (i) the site was of high antigenic index so that PEG attachment could reduce immunogenicity; (ii) it located in protrusion regions of the protein surface to enable efficient coupling; and (iii) it was away from the catalytic center and interaction site so that *N*-glycosidase activity was least impaired. A total of nine antibody epitopes were predicted using ElliPro serve [20]. Among these epitopes, two sites, namely Lys-78 and Lys-264, were selected for modification in accordance with the above criteria (Figure 1).

### 2.2. PEGylated Maize RIP Variants only Lost Half of the Ribosome-Inactivating Activity

**Figure 2:**
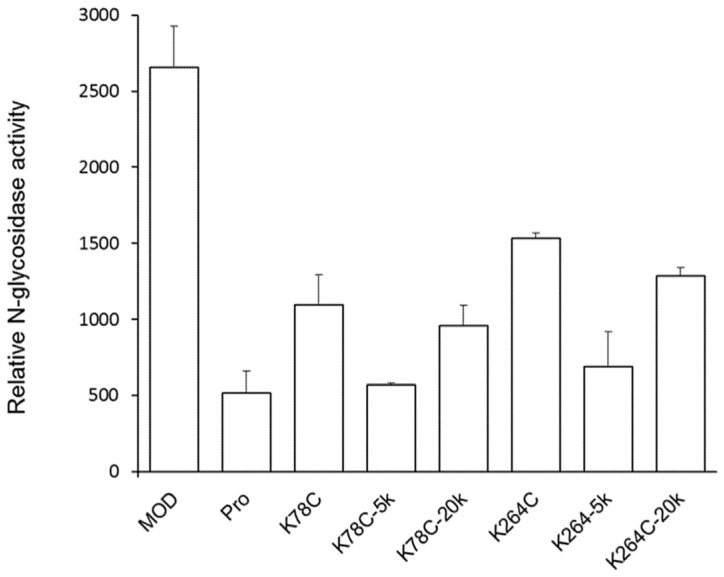
Relative *N*-glycosidase activity of maize RIP variants, mutants and PEGylated variants in human T lymphocyte (C8166). All the proteins contain the His-tag and TAT sequence at the *N*-termini. C8166 cells in log-phase growth were seeded at density of 1 × 10^6^ cell/well on a six-well plate and treated with 5 µM protein samples. After incubation at 37 °C for 6 h, cells were harvested for RNA isolation, cDNA synthesis and *N*-glycosidase activity was determined by qPCR using primers that target the modified site as previously described [8]. The relative *N*-glycosidase activity was calculated as the relative amount of depurinated rRNA of protein-treated cells against that of untreated cells. Data are presented as mean ± SD (*n* = 3).

MOD-PEG conjugates were evaluated for *N*-glycosidase activity on the human T lymphocyte C8166 cell line using the quantitative PCR method. The active form His-TAT-MOD exerted over a five-fold stronger SRL-depurinating effect than the precursor His-TAT-Pro (containing the internal inactivation loop) (Figure 2). Compared to the wild-type, non-PEGylated mutants had the activity decreased for approximated 50% and PEG conjugates had the bioactivity further reduced, with MOD-PEG_20k_ displayed a better depurinating effect than MOD-PEG_5k_.

### 2.3. PEG_20k_-Conjugated Maize RIP Variants Significantly Prolonged Circulating Half-Life in Rats

**Figure 3:**
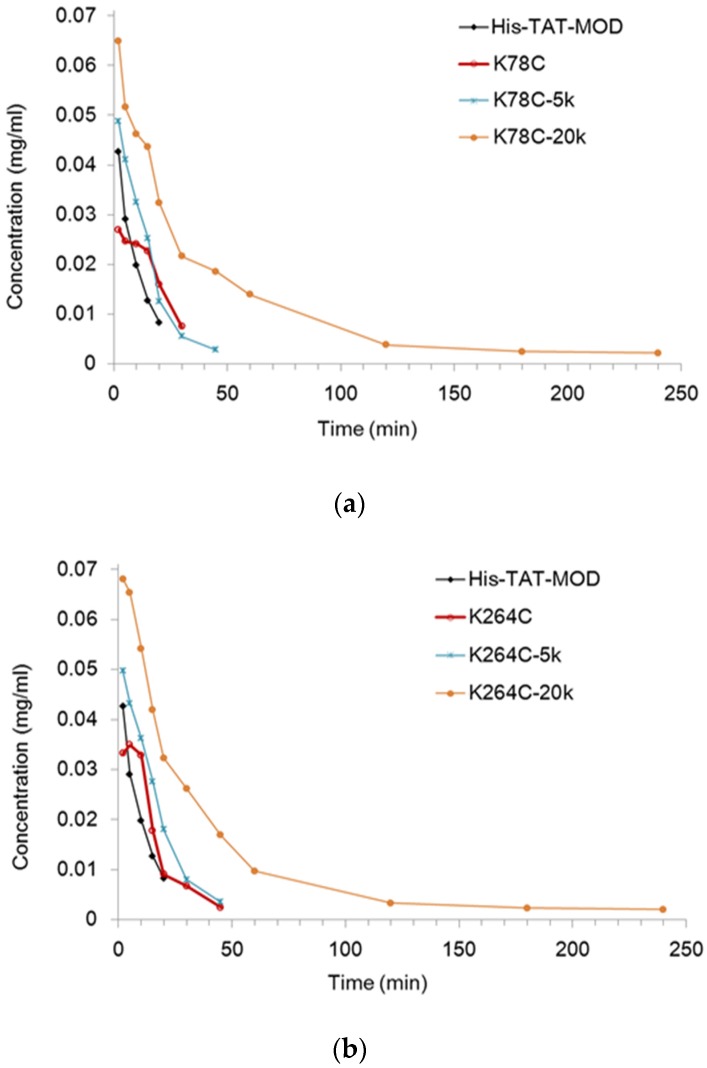
Plasma concentration–time profiles of maize RIP variants in rats. MOD-PEG_20k_ conjugates were detected 4 h after dosing whereas the non-PEGylated variants and MOD-PEG_5k_ conjugates had their concentrations below the detection limit within 1 h. (**a**) Plasma concentration–time profiles of K78C mutant and its PEGylated variants. (**b**) Plasma concentration–time profiles of K264C mutant and its PEGylated variants.

The pharmacokinetics of PEGylated maize RIP variants were examined in rats administered with a single intravenous injection of protein samples. To evaluate the plasma concentration of the variants, the corresponding antigen level was measured by ELISA (Figure 3). In both MOD-K78C and MOD-K264C, the PEG_20k_ conjugates could be detected 4 h after dosing whereas all other variants had their plasma levels below the detection limit within 1 h and could not have the concentration estimated. Table 1 lists the pharmacokinetic parameters calculated using WinNonlin software (version v3, Certara, Princeton, NJ, USA). As shown, MOD-PEG_5k_ conjugates had comparable plasma half-lives as the corresponding unmodified variants whereas coupling with PEG_20k_ extended the plasma half-life by five-fold for MOD-K78C and 17-fold for MOD-K264C, respectively. The more prominent prolonging effects of MOD-PEG_20k_ conjugates on in vivo half-life are likely attributed to the larger size of the attached PEG which makes the variants more resistant to degradation or clearance.

### 2.4. PEG_20k_-Modified Maize RIP Variants Elicit Weak Immune Responses in Mice

**Figure 4:**
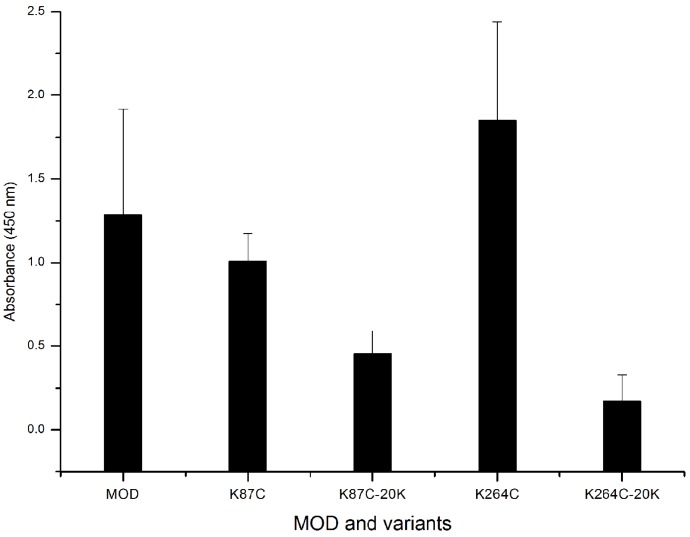
ELISA detection of specific IgG levels in mice serum. C57BL/6N inbred mice six to eight weeks old were randomly assigned into groups, and immunized with wild-type MOD (His-TAT-MOD), MOD mutants and PEGylated variants. ELISA assays were carried out at a 500-fold dilution of serum samples and each sample was repeated in triplicate.

Mice immunized with maize RIP variants were detected for IgE/IgG antibody generation to assess the effect of PEGylation on immunogenicity. By ELISA assays, no signal of maize RIP–specific IgE was found in treated animals whereas IgG was detected and shown in Figure 4. Both variants, K78C and K264C, showed subtle differences in IgG levels compared to the wild-type MOD. However, the variants modified with PEG_20k_ conjugates were shown to trigger lower IgG levels compared to the wild-type MOD and MOD variants. The K264C-PEG_20k_ conjugate gave the most significant decrease in IgG level.

## 3. Discussion

With high cytotoxicity and potent antiviral and anti-tumor effects, RIPs have been investigated as standalone molecules or as immunotoxins [3,11,21,22]. Even though the anti-HIV mechanism is still unclear, it has reported that several classical type I and II RIPs, such as ricin A chain [23], GAP31 [24], DAP30 [25], MAP30 [26], pokeweed antiviral protein (PAP) [10] and TCS [27], possess anti-HIV activity by inhibiting viral replication in vitro and in vivo. Our previous HIV-inhibitory activity studies showed the active maize RIP can suppress viral replication in acutely HIV-1 infected C8166 cells [8]. Recently, we assessed the anti-HIV effect of maize RIP on simian immunodeficiency virus (SIV)- and chimeric simian/human immunodeficiency viruses (SHIV)-infected macaque peripheral blood mononuclear cells (PBMC) and tested the antiviral activity of maize RIP in SHIV 89.6–infected Chinese rhesus macaque model [9]. We showed that the active recombinant maize RIP, His-TAT-MOD, can enhance PBMC cell survival and reduce the viral load in SHIV-infected macaque cells, suggesting His-TAT-MOD is a promising anti-HIV agent.

However, RIP-derived products have shortcomings such as a short plasma half-life and adverse immunogenic responses. Here, Lys-78 and Lys-264 were converted to cysteine and coupled with PEG_5k_ or PEG_20k_ to improve the above pharmacological properties. The resultant conjugates were shown to have enhanced blood circulation (Figure 3 and Table 1) and lower immunogenicity (Figure 4). We also found that MOD-PEG_5k_ conjugates showed a limited prolonging effect, whereas MOD-K78C- and MOD-264C-PEG_20k_ conjugates had the half-lives increased for five- and 17-fold, respectively. A large-sized molecule has reduced plasma clearance via renal filtration or proteolytic degradation [12]. The coupling of highly hydrophilic PEG may also facilitate the dissolution of conjugates within circulation. 

RIPs are toxins that depurinate a specific adenine nucleotide at the 28S rRNA of the large ribosomal subunit, resulting in the ceasing of protein synthesis. The RIP activities of the PEGylated products were assessed by the *N*-glycosidase activity on the T lymphocyte C8166 cell line. Despite the PEGylation sites, K78 and K264, employed in this study being remote from the highly conserved catalytic center and putative interaction site with the ribosome, it was found that the non-PEGylated variants and MOD-PEG conjugates displayed decreased RIP activity compared to wild-type MOD (Figure 2). A possible reason may be that such a modification has obstructed the access of the RIP to the ribosome.

Though PEG conjugation reduces biological activity, this loss is often compensated by the prolonged plasma half-life and in turn gives enhanced in vivo efficacy [19,28]. In this case, compared to the unconjugated MOD, the pharmacological effectiveness of MOD-K264C-PEG_20k_ showed an obvious advantage to prolong the half-life to five-fold while only half the *N*-glycosidase activity was lost.

**Figure 5:**
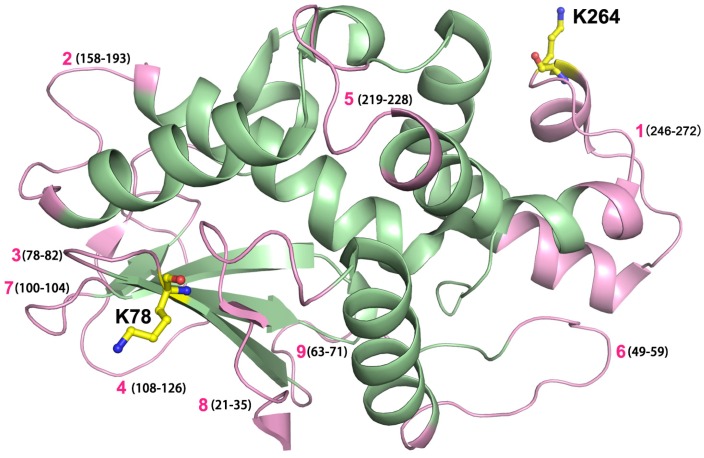
Predicted antibody epitopes in active form of maize RIP, MOD. Potential antibody epitopes in maize RIP predicted by the ElliPro program. These epitopes are labeled individually and the amino acid numbers are marked in brackets. The MOD molecule (PDB: 2PQI) was shown in pale green, and predicted nine epitopes are labeled in pink color. Two lysine residues, as labeled yellow sticks K78 and K264 located in epitopes 1 and 3 (pink), were mutated respectively in this study to attain cysteine-specific PEGylation.

Modification with inert polymers covers the protein surface to mediate the shielding effect over antibody epitopes and PEGylation has been employed to reduce the immunogenicity of several RIPs, as exemplified by the type I trichosanthin [19] and MAP30 [29]. In this study immunogenicity of maize RIP and its PEG conjugates was examined in C57BL/6N inbred mice and no detectable IgE production was found upon immunization. On the other hand, IgG levels were determined and the results showed that in both K78 and K264 cases, the lysine-to-cysteine mutation dose did not decrease the IgG response of the MOD. However, PEG_20k_ coupling led to a substantial decline of the IgG level (Figure 4), suggesting K78 and K276 are located in two important antigenic epitopes of MOD, and thus PEG modification of these two residues can effectively block the immunogenicity of MOD. Figure 5 summarizes the locations of all possible antibody epitopes in MOD predicted by ElliPro and PEG attachment at K78C and K264C could help mask the protruding antigenic regions from antibody recognition, thereby causing less IgG response. In summary, with a series of biochemical and in vivo assays, we have shown that coupling with PEG helps enhance the plasma circulating life and alleviate the immune response elicited by MOD, suggesting the resultant PEGylated variants are of improved therapeutic value. Our study also benefits the rational engineering design and development of effective RIP-related biomedicine.

## 4. Materials and Methods

### 4.1. Computer Modeling for PEGylation Sites

Conjugation with PEG took place specifically at cysteine residue where the polymer was covalently linked to the protein through thioether bond formation. The ElliPro method was employed to predict B-cell epitopes in maize RIP for PEGylation [30]. Computational analysis identified two lysines of significant antigenic index, namely K78 and K264, in the amino acid sequence of maize RIP which were then selected for PEG-coupling.

### 4.2. Cloning, Expression and Purification of Maize RIP Variants

To attain mono-PEGylation, the native cysteine residues in maize RIP, C51 and C206, were substituted with serine, followed by the lysine-to-cysteine mutations at selected modification sites. Two maize RIP variants, namely MOD-K78C and MOD-K264C, were constructed using Phusion DNA polymerase (Finnzymes) and primers containing the desired modifications (Table 2) with the recombinant plasmid His-TAT-MOD-pET3a as template. DNA products were cloned into expression vector pET3a and sequenced to confirm correct mutagenesis. All variant proteins also contain His-tag and TAT sequence at the *N*-termini, and they were expressed, purified, and stored in the same manner as previously described [31].

### 4.3. Preparation of PEGylated Variants

Cysteine-specific PEGylation was carried out by incubating the maize RIP variants with methoxy PEG (5 or 20 kDa)-maleimide reagents (Nanocs) in 20 mM phosphate buffer, 10 mM EDTA, pH 6.5 overnight at 4 °C [12]. 

MOD-PEG_5k_ reaction mixture with buffer exchanged to 20 mM phosphate buffer, 1.5 M (NH_4_)_2_SO_4_ was loaded to a 5 mL HiTrap Phenyl High Performance column (GE Healthcare Biosciences, Pittsburgh, PA, USA) for hydrophobic interaction chromatography. Elution was carried out with 1.05–0.15 M (NH_4_)_2_SO_4_ linear gradient and fractions with MOD-PEG_5k_ conjugate were pooled and concentrated.

MOD-PEG_20k_ conjugate was purified stepwise using ion-exchange and size-exclusion chromatography. MOD-PEG_20k_ reaction mixture was first loaded to a 5 mL HiTrap DEAE Fast Flow column (GE Healthcare) equilibrated with 20 mM NaOAc, pH 6.5 and eluted with a 0.5–1.0 M NaCl linear gradient. Fractions of higher purity were pooled and loaded to Superdex75 (GE Healthcare Biosciences, Pittsburgh, PA, USA) equilibrated with 20 mM phosphate buffer, 0.5 M NaCl, pH 6.5 for further isolation. Fractions with pure MOD-PEG_20k_ were identified by SDS-PAGE.

All variants were buffer-exchanged to 20 mM phosphate buffer, 200 mM NaCl, 5% glycerol, pH 7.4 and stored at −80 °C.

### 4.4. Evaluation of Sarcin-Ricin Loop Depurination Activity

Human T lymphocyte cell line (C8166) was obtained from AIDS Reagent Project, Medical Research Council, UK and maintained in RPMI-1640 medium supplemented with 10% fetal bovine serum (Invitrogen, Carlsbad, CA, USA). C8166 cells in log-phase growth were seeded at density of 1 × 10^6^ cell/well on six-well plate and treated with 5 µM protein samples. After incubation at 37 °C for 6 h, cells were harvested for RNA isolation and cDNA synthesis as previously described [8]. Degree of SRL depurination was estimated by quantitative real-time PCR method [32] using 7500 Fast Real-Time PCR System (Applied Biosystems, Foster, CA, USA) and Power SYBR Green PCR Master Mix Kit (Applied Biosystems, Foster, CA, USA). The relative amount of depurinated rRNA was calculated as nucleic acid estimated by test primers divided by that estimated by control primers. The relative SRL depurination activity was determined as the relative amount of depurinated rRNA of treated cells against that of untreated cells.

### 4.5. Pharmacokinetics Study

Sprague-Dawley rats weighed 230–250 g were supplied by the Laboratory Animal Services Centre of The Chinese University of Hong Kong and experiments were performed in accordance with the CUHK Basic Principles and Guidelines (License No. (13-351)IN DH/HA&P/8/2/1 PT.31, 10 September 2013, Department of Health, Hong Kong SAR). Animals were fasted overnight with free access to water. The right jugular vein of rat was cannulated with a polyethylene tube (Braintree Scientific, Inc., MA, USA) under ketamine/xylazine (80/20 mg/kg) induced anesthesia. Prior to protein injection, rat was allowed to recover for 1 h. The cannulated animal was administered with maize RIP variant at dose of 4.5 mg/kg intravenously, followed by injecting 0.5 mL blank rat blood and then 0.5 mL heparinized normal saline (25 I.U./mL) to rinse the catheter. The blood samples, approximately 0.4 mL, were collected into heparin-rinsed tubes at 2, 5, 10, 15, 20, 30, 45, 60, 120, 180 and 240 min post dosing. Plasma was isolated by centrifugation of blood at 13,000× *g* for 5 min.

ELISA was carried out to estimate the concentration of MOD or variant in plasma for in vivo half-life determination. In brief, a 96-well ELISA plate (Thermo Fisher Scientific, Waltham, MA, USA) was pre-coated with polyclonal rabbit anti-MOD antibody in 0.05 M sodium carbonate/bicarbonate buffer, pH 9.6 overnight at 4 °C. The plate was then rinsed three times with washing buffer (PBS with 0.5% Tween 20) and blocked with 5% non-fat milk at 37 °C for 2 h. Diluted plasma samples were added and incubated at 37 °C for 2 h. After washing, biotin-labeled anti-MOD antibody was applied for detection followed by streptavidin-horseradish peroxidase conjugate (Invitrogen, Carlsbad, CA, USA). Finally, 3,3′,5,5′-tetramethylbenzidine (TMB) substrate solution (BD Bioscience, Bedford, MA, USA) was added and incubated at room temperature for 10 min. The reaction was terminated by adding 1 M H_2_SO_4_ and OD_450nm/630nm_ was measured using an ELISA plate reader. Pharmacokinetic parameters were calculated by WinNonlin software (version 3, Certara, Princeton, NY, USA).

### 4.6. Immunogenicity Assay

Immunization and blood collection of mice were conducted at Guangdong Medical Laboratory Animal Centre, Foshan, China. C57BL/6N inbred mice of 6-8 week old were randomly assigned into groups of six. Wild-type or PEGylated variants were administered subcutaneously at the back with 10 µg in complete Freund’s adjuvant on Day 0. Sampling for IgE detection was carried out on Day 10. Booster injection was given with incomplete Freund’s adjuvant on Day 21. Sampling for IgG detection was performed 7 day after booster injection by retrobulbar puncture. Blood samples were centrifuged instantly right after collection and the isolated sera were stored at −80 °C.

IgE and IgG specific for maize RIP were detected by ELISA method. In brief, a 96-well ELISA plate (Thermo Fisher Scientific, Waltham, MA, USA) was pre-coated with antigen in 0.1 M sodium carbonate/bicarbonate buffer, pH 9.6 overnight at 4 °C. The plate was then washed and blocked with 5% non-fat milk at 37 °C for 2 h. Next, diluted serum samples were added for incubation at 37 °C for 2 h. After washing, the specific secondary detecting antibody (Goat anti-Mouse IgE Secondary Antibody-HRP conjugates, Goat anti-Mouse IgG (H + L) Secondary Antibody-HRP conjugates (Thermo Fisher Scientific, Waltham, MA, USA) was added and incubated at 37 °C for 2 h, followed by TMB substrate solution (BD Bioscience, Bedford, MA, USA). After termination, OD_450nm/630nm_ was measured with an ELISA plate reader.

## Figures and Tables

**Table 1:** Statistic and pharmacokinetic parameters of MOD and variants.

Treatment	AUC_0-t_ (mg min/mL)	T_1/2_ (min)	Fold Increased Compared to Non-PEGylated Form
His-TAT-MOD	0.46	8.0	-
MOD-K78C	0.59	9.4	-
MOD-K78C-5k	0.81	9.8	1.04
MOD-K78C-20k	2.65	47.6	5.06
MOD-K264C	0.68	10.1	-
MOD-K264C-5k	0.93	10.1	1
MOD-K264C-20k	2.57	171.1	16.94

All parameters were calculated by the WinNonlin software. AUC_0-t_: the area under the curve from the time of dosing to the time of the last observation; T_1/2_ is the time required for a quantity to reduce to half its initial value.

**Table 2:** List of primers and their corresponding sequences used for constructs. The mutated amino acid is underlined.

Primer	Sequence (5′-3′)
MOD-C15S-F	GTGATCAAACACTCTACCGACC
MOD-C15S-R	GGTCGGTAGAGTGTTTGATCAC
MOD-C206S-F	GTGGTCATGGTGTCTGAGGGGCTG
MOD-C206S-R	CAGCCCCTCAGACACCATGACCAC
MOD-K78C-F	ACAGAGCTCTGTACTAGGACC
MOD-K78C-R	GGTCCTAGTACAGAGCTCTGT
MOD-K264C-F	GACATGCAGTGTCTTGGCATC
MOD-K264C-R	GATGCCAAGACACTGCATGTC
